# An integrative bioinformatics investigation and experimental validation of critically involved genes in high-grade gliomas

**DOI:** 10.1186/s13000-022-01253-0

**Published:** 2022-09-24

**Authors:** Reza Ahmadi-Beni, Shirin Shahbazi, Alireza Khoshnevisan

**Affiliations:** 1 Department of Medical Genetics, Faculty of Medical Sciences, Tarbiat Modares University, Jalal Al Ahmad highway, Tehran, 14115-111 Iran; 2 Department of Neurosurgery, Shariati Hospital, Tehran University of Medical Sciences, Tehran, Iran

**Keywords:** Adult glioma, Integrated bioinformatics, GEO data, Differentially expressed genes

## Abstract

**Background:**

Lack of knowledge around underlying mechanisms of gliomas mandates intense research efforts to improve the disease outcomes. Identification of high-grade gliomas pathogenesis which is known for poor prognosis and low survival is of particular importance. Distinguishing the differentially expressed genes is one of the core approaches to clarify the causative factors.

**Methods:**

Microarray datasets of the treatment-naïve gliomas were provided from the Gene Expression Omnibus considering the similar platform and batch effect removal. Interacting recovery of the top differentially expressed genes was performed on the STRING and Cytoscape platforms. Kaplan–Meier analysis was piloted using RNA sequencing data and the survival rate of glioma patients was checked considering selected genes. To validate the bioinformatics results, the gene expression was elucidated by real-time RT-qPCR in a series of low and high-grade fresh tumor samples.

**Results:**

We identified 323 up-regulated and 253 down-regulated genes. The top 20 network analysis indicated that PTX3, TIMP1, CHI3L1, LTF and IGFBP3 comprise a crucial role in gliomas progression. The survival was inversely linked to the levels of all selected genes. Further analysis of RNA sequencing data indicated a significant increase in all five genes in high-grade tumors. Among them, PTX3, TIMP1 and LTF did not show any change in low-grade versus controls. Real-time RT-qPCR confirmed the in-silico results and revealed significantly higher expression of selected genes in high-grade samples compared to low-grade.

**Conclusions:**

Our results highlighted the role of PTX3 and TIMP1 which were previously considered in glioma tumorigenesis as well as LTF as a new potential biomarker.

## Introduction

Gliomas, the most prevalent and aggressive type of brain tumors, are classified by the world health organization (WHO) as grade I-IV according to clinical and histopathological characteristics. Grade I comprise benign and relatively low-risk gliomas. Grade II is known as low-grade gliomas (LGG), displaying well-differentiated tumor cells with a better prognosis. WHO grade III-IV classifications include high-grade gliomas (HGG). They are characterized by undifferentiated cells and consist of anaplastic gliomas (grade III) and glioblastoma multiforme (GBM) (grade IV) [1].

LGG, which generally affects young adults with an average age of 40, eventually leads to GBM and death with a survival of fewer than ten years. In recent years, a new classification has been proposed by the WHO regarding the status of the isocitrate dehydrogenase (IDH) gene. Tumors bearing mutations in IDH are sub-classified into secondary GBM, indicating that they are originated from a LGG tumor [2]. However, about 90% of GBM are de novo cases with poor prognosis and a median survival of around one year [3].

Despite the significant advances in understanding gliomas genomic alterations, the molecular basis of HGG remains to be explored. The high molecular heterogeneity of gliomas could explain the complexity of its prognosis prediction [4].

The clinical aggressiveness within gliomas has been explored by the development of high‐throughput technologies [5]. Glioma-related bioinformatics studies mostly conducted by using the online available microarray datasets such as Gene Expression Omnibus (GEO) [6], The Cancer Genome Atlas (TCGA) [7] and ArrayExpress [8]. However, shortcomings in sample selection, including *de-novo* versus secondary GBM and treatment-naïve versus treatment-experienced have led to heterogeneous outcomes of differentially expressed genes (DEGs). Diverse data processing methods and technological platforms could be mentioned as additional confounding factors and may result in controversies. Integrated bioinformatics and co-expression networks analysis assist network-based gene screening and strengthen the statistical analysis [9].

In the present study, by the integrative and network-based approach, we analyzed DEGs between adult LGG and HGG. We also determined the involved signaling pathways and potential molecular interactions that are significantly associated with WHO grading and prognosis. DEGs revealing major alterations in their network interactions between LGG and HGG were selected for further validation in patient’s tumor samples.

## Materials and methods

### Data integration, batch effect removal

GSE4290, GSE15824, GSE19728, GSE43378, and GSE51062 microarray expression datasets were collected from the GEO database (http://www.ncbi.nih.gov/geo) using the GEO query package of R software. All of the selected microarrays had been performed on treatment-naïve adult gliomas by the GPL570 detection platform. The datasets comprised a total of 345 samples, including HGG, LGG, glioma cell lines, and normal brain tissues. The samples of the normal brain, cell lines, unknown subtypes and secondary or recurrent tumors were excluded from the study and ultimately 301 treatment-naïve adult samples including 238 HGG and 63 LGG were recruited for the subsequent analysis. The average expression value of all mapped probe sets was considered for each gene. The preprocessed expression datasets were merged by the R program into one global expression matrices according to the gene symbol. Using the combat method in R, the technical heterogeneity or batch effect was removed across the datasets.

### DEGs identification

The limma package of R was utilized to determine the DEGs between LGG and HGG in a linear model. To decrease the false positive rate and false discovery rate the cut-off criteria of |log2FC|> 1 and an adjusted *P*-value < 0.001 were selected. The results were presented in a volcano plot of log-fold changes on the X-axis versus adjusted statistical significance on the Y-axis.

### Functional annotation and pathway analyses

Gene Ontology (GO) enrichment analysis and pathway enrichment analysis based on and Kyoto Encyclopedia of Genes and Genomes (KEGG) pathways were conducted by Database for Annotation, Visualization, and Integrated Discovery (DAVID) online tool (https://david.ncifcrf.gov/). The *p*-value ˂0.05 was considered statistically significant.

### Construction of protein–protein interaction network

The STRING online tool (https://string-db.org) [10] was constructed to generate the protein–protein interaction (PPI) network of the top 20 up and down-regulated DEGs. To recognize the target genes, the PPI network was visualized by the network analyzer in the Cytoscape software (version 3.8.2, http://www.cytoscape.org/) [11].

### Top DEGs validation by glioma RNA sequencing data

DEGs with significant protein–protein interactions were selected for further assessments through the Gene Expression Profiling and Interactive Analyses (GEPIA) online RNA sequencing-based database (http://gepia.cancer-pku.cn) [12]. This database represents the gene expression variations in cancer samples based on data from TCGA and The Genotype-Tissue Expression (GTEx). Kaplan–Meier and Cox proportional hazard analysis were conducted to check the survival rate of glioma patients by subgrouping into the low and high expression of selected genes.

By the comparison between GBM, LGG and healthy tissue, the expression levels of selected genes were presented as mean (standard deviation) in boxplots. A *p*-value ˂0.01 was considered statistically significant.

### Patient’s samples collection

To validate the *in-vitro* expression of selected hub genes, we obtained a series of fresh LGG and HGG samples. The research was approved by the Ethics Committees of Tarbiat Modares University and performed under the Helsinki declaration of 1975, as revised in 2013. Following informed consent, 11 LGG and 11 HGG tissue samples were collected from Shariati hospital, Tehran, Iran. A part of the tumor samples was sent for pathological analysis.

### Gene expression analysis

Total mRNA of the samples was extracted by the All-In-One kit (Bio Basic, Canada). The reverse transcription‑quantitative polymerase chain reaction (RT-qPCR) was performed on extracted mRNA as described before [13]. For normalizing target genes, we selected eukaryotic translation initiation factor 2B subunit alpha (EIF2B1) as the reference gene [14]. By evaluation on GEPIA, EIF2B1 showed higher expression and lower fluctuation compared to other recommended reference genes such as C-terminal binding protein 1 (CTBP1), mitochondrial ribosomal protein L9 (MRPL19) and TATA box-binding protein (TBP).

The primer sequences of selected and reference genes were as follow: forward PTX3 5'-TGCATCTCCTTGCGATTCTGT-3', reverse PTX3 5'-AGCTTGTCCCATTCCGAGTG-3', forward CHI3L1 5'-ATGATGTGACGCTCTACGGC-3', reverse CHI3L1 5'-ACTCTGGGTGTTGGAGGCTA-3', forward IGFBP3 5'-CAGAATATGGTCCCTGCCGTAG-3', reverse IGFBP3 5'-TTTGGAAGGGCGACACTGC-3', forward TIMP1 5'-GCTTCTGGCATCCTGTTGTTG-3', reverse TIMP1 5'-GGTGGTCTGGTTGACTTCTGG-3', forward LTF 5'-ATCGCCCTGGTGCTGAAAG-3', reverse LTF 5'- GGGTCACTGCTTTGTTGGGA-3', forward IEF2B1 5'-CGGACGTTGCTGGAGTTCTT-3', reverse EIF2B1 5'-AGGCAAGACTGATGAAGCGG-3'.

To perform statistical analysis, the Mann–Whitney test was applied for the comparison of the groups. GraphPad Prism8 was used to generate plots signifying the levels of gene expression. A difference of *p*-value ˂0.05 was considered statistically significant.

## Results

### Identification of DEGs in merged datasets

**Fig. 1:**
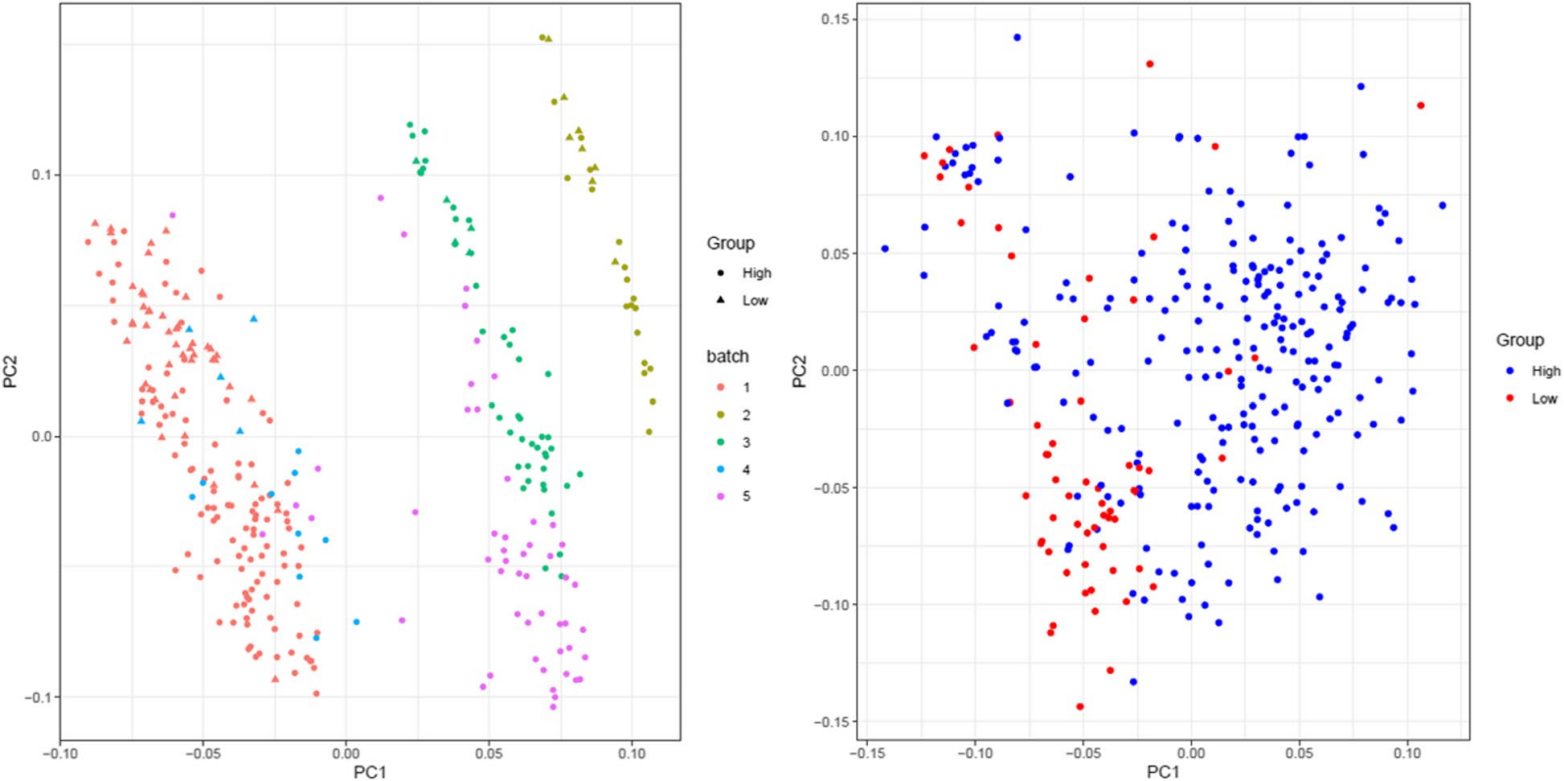
PCA scatter plot based on gene expression profiles in 301 samples from 5 datasets. Left: Merged datasets before batch effect correction; datasets are specified with different colors. LGG and HGG samples are shown with circle and triangle symbols, respectively. Right: Merged datasets after batch effect correction; blue and red dots are shown HGG and LGG samples, respectively

**Fig. 2:**
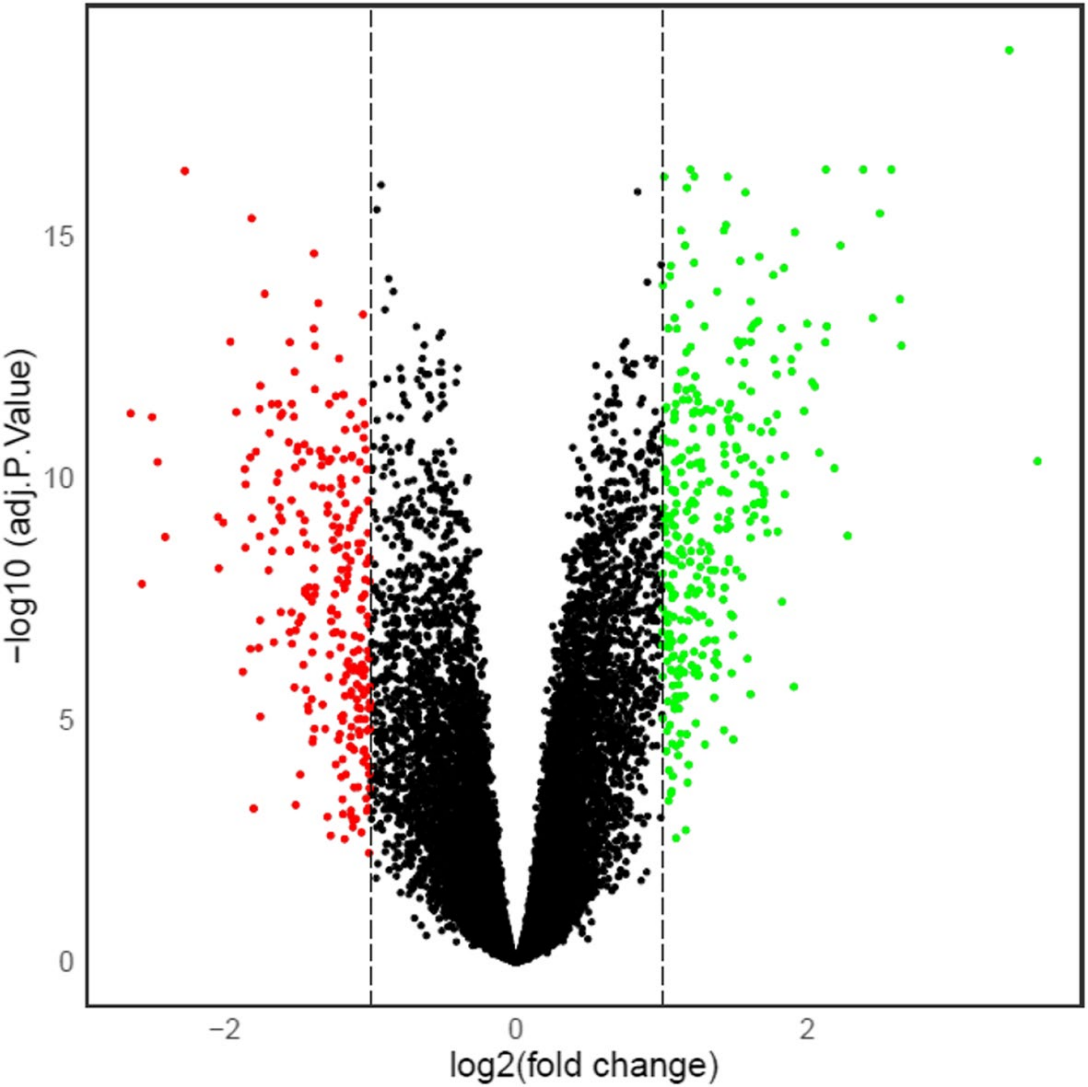
Volcano plot visualizing the DEGs in a total of 22,189 genes; green points represent 323 upregulated and red represents 253 down-regulated genes

The principal component analysis (PCA) as an unsupervised clustering approach was constructed to classify LGG and HGG samples before and after batch effect corrections. The proper discrepancy between LGG and HGG samples was detected before adjusting the batch effect. Admissible clustering was reached following batch effect removal (Fig. 1). According to the PCA results, five samples were considered an outlier and were removed from the subsequent analysis. By merging the datasets, a total of 22,189 common genes were considered for further analysis. A total of 576 DEGs were identified comprising 323 upregulated and 253 down-regulated genes. The results were illustrated as a volcano plot in Fig. 2, in which the up and down-regulated genes are represented in green and red dots, respectively.

### Functional annotation and pathway analyses

**Fig. 3:**
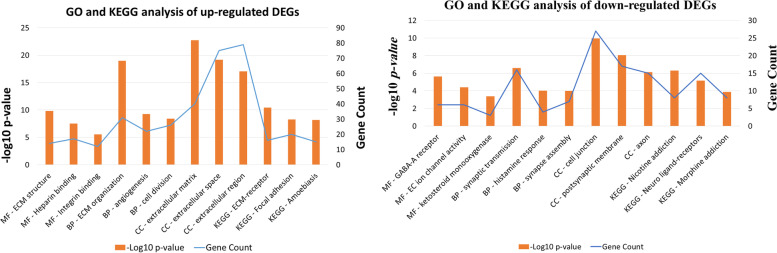
Top three pathways analysis of molecular function (MF), biological process (BP) and cellular components (CC) of GO, as well as KEGG analysis results. Left; up-regulated DEGs, Right; down-regulated DEGs

To discover the biological functions of the identified DEGs, the up-regulated and down-regulated genes were separately enriched in three categories regarding the molecular function, biological process, and cellular component. The up-regulated DEGs in the molecular function class were mainly enriched in extracellular matrix (ECM) structural constituent, heparin, integrin and PDGF binding. These DEGs were also significantly enriched in the ECM organization, angiogenesis and cell division in the biological process. In the cellular component category, they were enriched in the ECM, extracellular space, and extracellular region. According to the KEGG pathway analysis, the up-regulated DEGs were frequently enriched in ECM-receptor interaction and focal adhesion (Fig. 3).

### Construction of PPI network

**Fig. 4:**
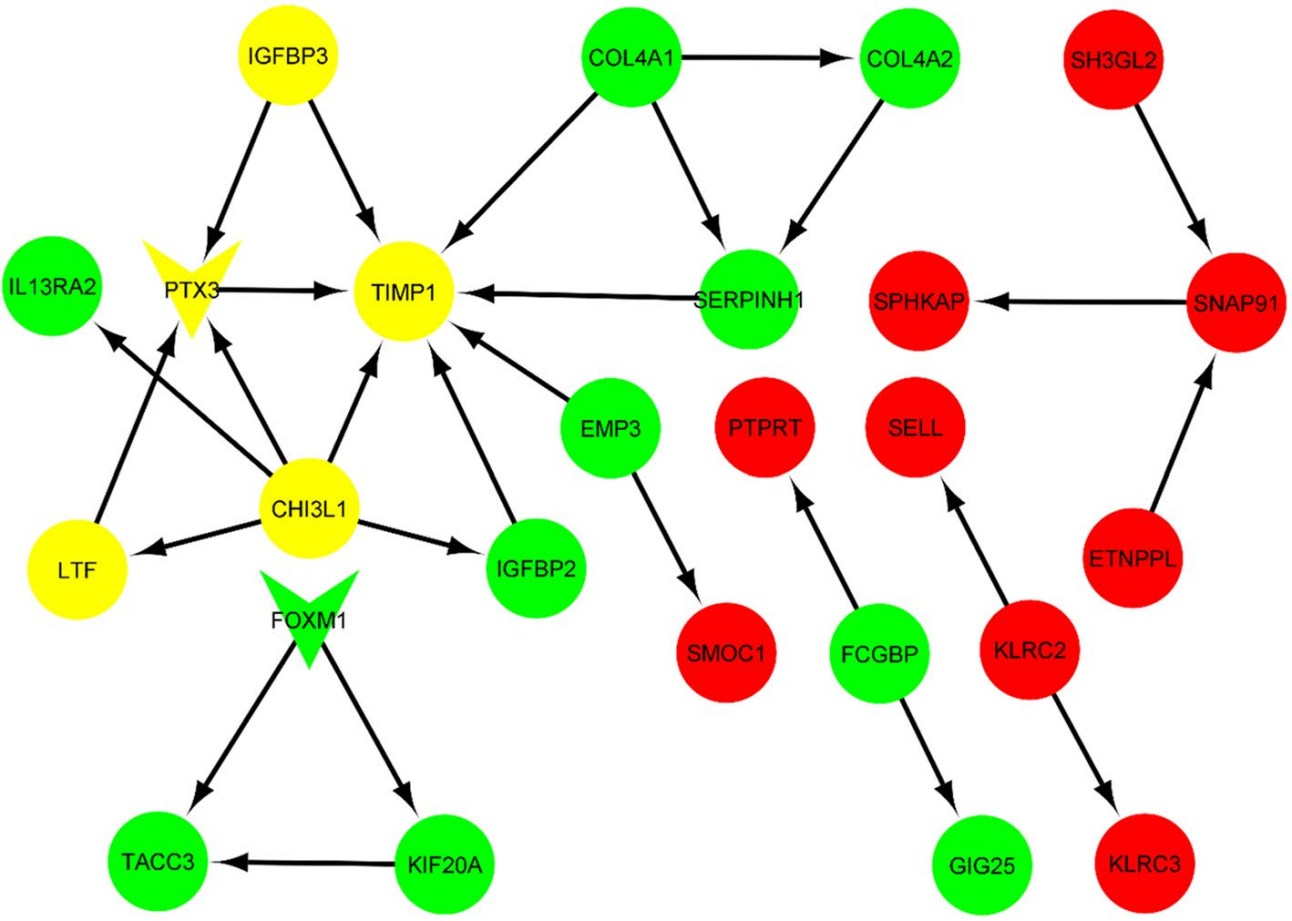
Protein–protein interaction network illustrated by Cytoscape software. The 25 genes of the top 20 DEGs interact with each other amongst them TIMP1, CHI3L1 and PTX3 have the most interactions. The green nodes represent upregulated genes, whereas nodes in red represent down-regulated genes. The Yellow circles contain upregulated hub DEGs with interactions to PTX3. Transcription factors are in triangular shape. The arrows represent the direction of interactions

The top 20 most significant up and down-regulated DEGs are shown in Table 1. The identified top 20 DEGs were mapped into the STRING database to find the interactions between their corresponding proteins. The construction of DEGs networks indicated significant interactions between pentraxin 3 (PTX3), chitinase-3-like protein 1 (CHI3L1), insulin-like growth factor-binding protein 3 (IGFBP3), tissue inhibitor of matrix metalloproteinases 1 (TIMP1) and lactotransferrin (LTF). As illustrated by Cytoscape software in Fig. 4, the 25 genes of the top 20 DEGs interact to each other, amongst them TIMP1, CHI3L1 and PTX3 have the most interface.

**Table 1:** Top 20 up-regulated and down-regulated differentially expressed genes between LGG and HGG

Gene Symbol	Log2FC	Adj.*P*.value
LTF	3.57	4.45E-11
IGFBP2	3.38	1.38E-19
TIMP1	2.57	4.09E-17
NNMT	2.64	1.80E-13
SERPINH1	2.63	1.97E-14
COL4A1	2.49	3.31E-16
CHI3L1	2.44	4.84E-14
EMP3	2.37	4.09E-17
IL13RA2	2.27	1.54E-09
VMP1	2.22	1.53E-15
SPOCD1	2.18	6.20E-11
SERPINA3	2.12	7.18E-14
COL4A2	2.12	4.09E-17
KIF20A	2.11	1.54E-13
PTX3	2.07	2.97E-11
FOXM1	2.04	1.27E-12
CA3	2.02	1.02E-12
IGFBP3	1.99	6.32E-14
FCGBP	1.97	4.06E-12
TACC3	1.93	1.91E-13
FSTL5	-2.64	4.56E-12
NTSR2	-2.56	1.50E-08
SFRP2	-2.49	5.42E-12
ETNPPL	-2.45	4.62E-11
KLRC2	-2.40	1.63E-09
CSMD3	-2.27	4.37E-17
SNAP91	-2.04	7.14E-09
CNTN3	-2.04	6.35E-10
HMP19	-2.00	8.28E-10
SPHKAP	-1.95	1.49E-13
MGAT4C	-1.91	4.28E-12
SMOC1	-1.86	6.48E-11
PTPRT	-1.85	1.33E-10
SH3GL2	-1.85	2.74E-09
ZNF488	-1.82	3.33E-07
KLRC3	-1.82	3.70E-11
FAM133A	-1.81	4.20E-16
INA	-1.81	6.74E-10
PRLHR	-1.78	2.82E-11
SELL	-1.76	3.68E-12

### In-silico validation of candidate genes

**Fig. 5:**
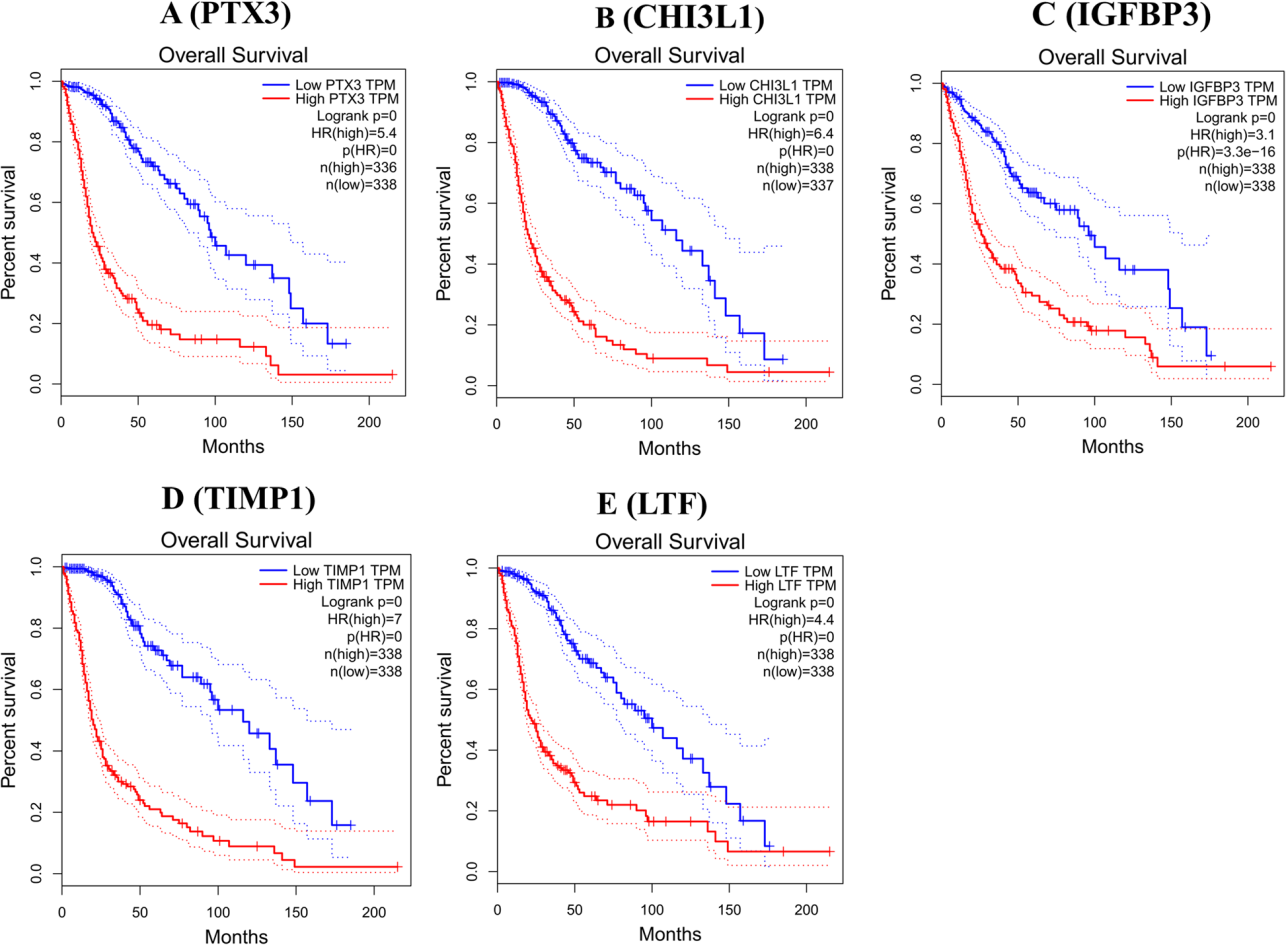
Kaplan–Meier survival curves using The Cancer Genome Atlas database validate the prognostic value of genes expressed in gliomas (blue—low risk; red—high risk)

The expression levels of candidate genes were further assessed in 163 HGG, 518 LGG and 207 healthy patients’ samples on the GEPIA web server. Initially, the correlation between gene expression and overall survival of glioma were assessed by Kaplan–Meier analysis. The patient’s survival in high mRNA expression levels of all five selected genes was less than that in the low expression group (Fig. 5).

**Fig. 6:**
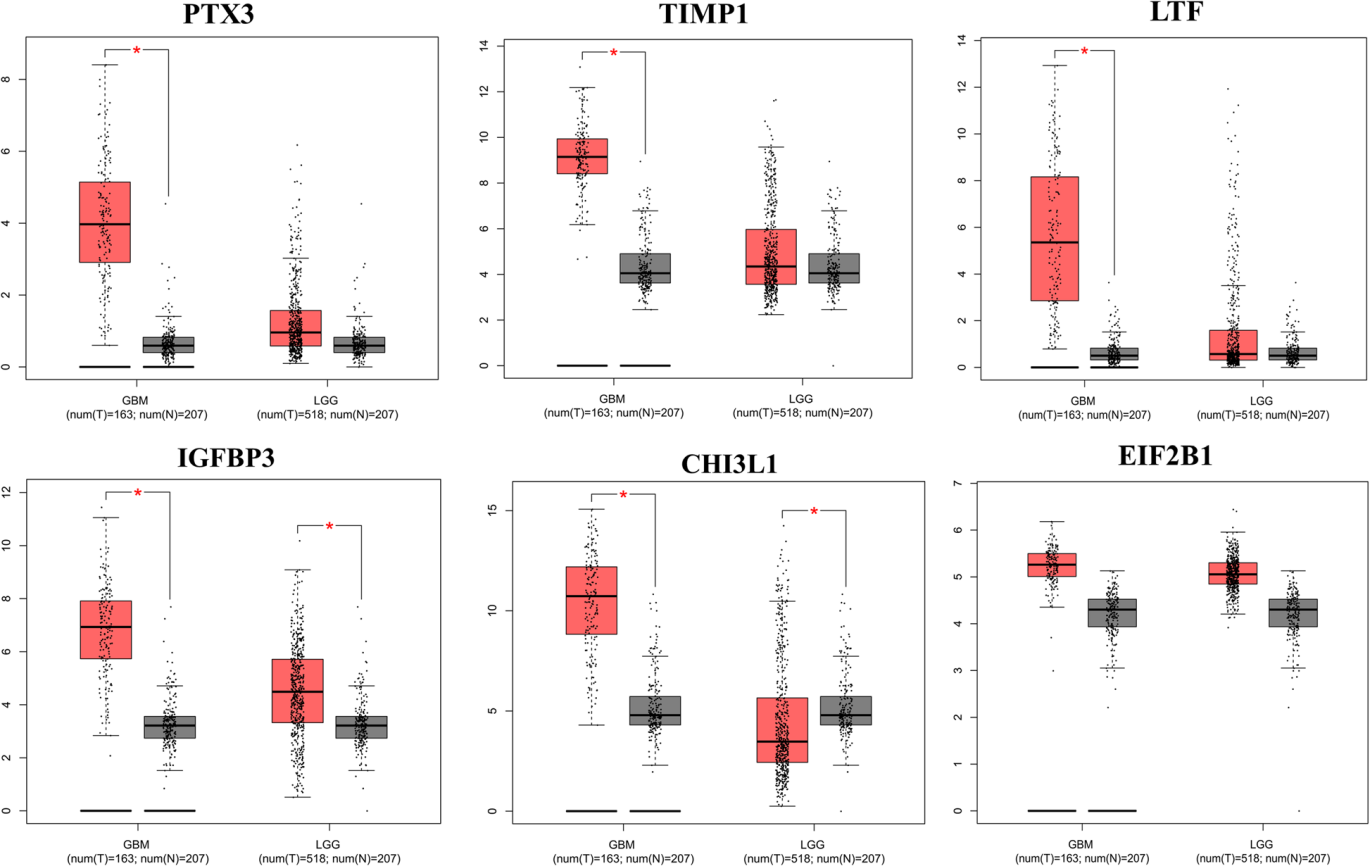
The GEPIA database results for the expression level of selected genes. The y axis represents log2(TPM + 1). TPM: transcripts per million

By dividing glioma patients into GBM and LGG groups, the expression level of candidate genes in each of these groups was compared with normal samples. The data indicated a significant increase in all five genes in GBM compared to LGG and control tissues. Gene expression levels of IGFBP3 and CHI3L1 significantly differed between LGG and normal brain tissue. However, the expression of PTX3, TIMP1 and LTF were not altered in LGG versus controls indicating their specificity in GBM pathogenesis (Fig. 6).

### Patient’s characteristics

The treatment-naïve glioma cases included eight male and fourteen female patients, with an average age of 52 ± 15 years old. The LGG samples consisted of five cases of diffuse oligodendroglioma (grade II) and six cases of diffuse astrocytoma (grade II), and the HGG samples included five cases of anaplastic astrocytoma (grade III) and six cases of glioblastoma multiform (grade IV).

### In vitro validation of gene expression by RT-qPCR

**Fig. 7:**
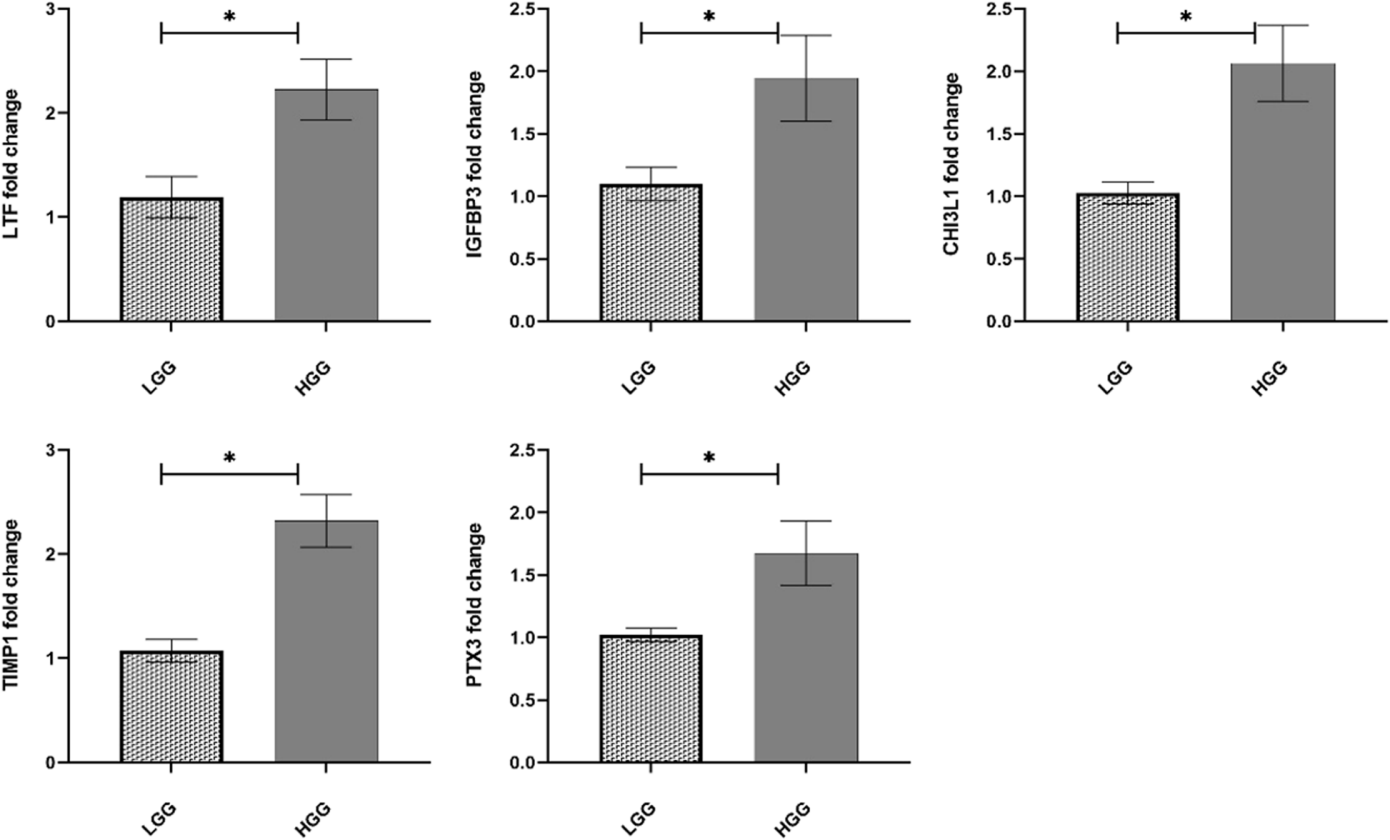
Mean relative expression of *LTF*, *IGFBP3*, *CHI3L1*, *TIMP1* and *PTX3* in HGG compared to LGG in patient samples

We conducted an RT-qPCR and confirmed the in-silico expression data of PTX3, IGFBP3, TIMP1, CHI3L1 and LTF. Normalized results of the expression, relative to the expression level of EIF2B1 mRNA indicated significantly higher expression of selected genes in HGG compared to LGG (*p*-value < 0.05) (Fig. 7). The highest fold changes of 2.33 and 2.23 were calculated for TIMP1 and LTF, respectively. CHI3L1 and IGFBP3 exhibited almost a twofold increase, while PTX3 disclosed fold change of 1.67 in HGG compared to LGG.

We could not detect significant expression differences related to patients’ age, sex, tumor size and/or tumor location.

## Discussion

HGG is considered a challenging subtype of brain tumors with a poor prognosis, aggressive features and lack of targeted therapies. Although many efforts have been made to develop effective treatments, tumor heterogeneity and unknown contexts remain limitations of discovered therapies [15]. Identification of the key genes and pathways involved in HGG pathogenesis is of particular importance to develop new therapeutic approaches. In recent decades, high throughput technologies produce huge data that are helpful for a well understanding of the molecular basis of tumorigenesis.

Here, we analyzed multiple expression array datasets to compare gene expression profiles of HGG versus LGG to provide the critically involved genes. Our results elucidated that the PTX3 gene acts as a master regulator in the HGG gene regulatory network by connectivity to four other DEGs. Compared to LGG, HGG showed increased PTX3 mRNA levels in patients’ tumor samples. PTX3 is mainly involved in immune responses and inflammation; however also plays various roles in multiple molecular mechanisms such as cell cycle, angiogenesis, metastasis, and genomic instability [16]. Dysregulation of PTX3 is associated with the early event in oncogenesis and may be acting as an initiating factor. By in vitro inactivation of PTX3, GBM cells migration and invasion were significantly eliminated [17].

Among the genes that showed a strong link with PTX, the TMP1 was already known to be associated with deteriorating and poor prognosis of glioma [18]. Aaberg-Jessen et al. investigated the prognostic potential of TIMP1 in combination with its cell surface binding protein, CD63, correlated to tumor grade and overall survival of glioma patients. However, no additional prognostic significance of TIMP1 was observed by including the CD63. They also analyzed DEGs comparing the highest and lowest CD63 mRNA level tumors using the TCGA dataset. They reported TIMP1, PTX3 and CHI3L1 among the upregulated DEGs involved in the regulation of cell survival and cellular movement [19].

In our study, we observed that mRNA expression of CHI3L1, also known as YKL-40, was significantly higher in HGG either by in silico or in vitro analysis. It is expressed as a mesenchymal marker in the most aggressive and challenging subtype of GBM associated with particularly poor outcome [20]. CHI3L1 is a secreted glycoprotein and is suggested as a promising serum marker for GBM diagnosis [21]. However, it has more diagnostic value in recurrent GBM than newly diagnosed patients [22].

Among up-regulated genes found in our study, LTF transcript was marked as the first ranked (Table 1); however, its promoting role in tumorigenesis is not evident. As an iron-binding protein, LTF has a wide spectrum of protective activities such as anti-microbial and immunity. Studies in nasopharyngeal carcinoma and prostate cancer suggested a tumor suppressor function for LTF [23, 24].

Using GEPIA, we found that the expression of PTX3, TIMP1 and LTF were specificity high in GBM, whereas they were not altered in LGG compared to normal controls. One of the suggested mechanisms that can be shared between their functions is the inflammatory process. Correlation to inflammatory in glioma tumorigenic processes has been demonstrated by different studies. Increased expression of PTX3 under the influence of transcriptional factor CCAAT / enhancer-binding protein delta (CEBPD) has been shown to inhibit the phagocytosis of dying neurons [25]. CEBPD also regulates the stemness of glioma cells by activating platelet-derived growth factor subunit A (PDGFA) expression due to inflammatory stimulation. As mentioned before, inflammation is one of the important processes in the development of GBM and response to treatment [26, 27]. It should be noted that LTF was previously reported as putative interactors of CEBPD [28].

We also found IGFBP3 among the top 20 DEGs that showed strong involvement with PTX3. IGFBP3 plays a direct role in signaling and cell growth pathways in association with insulin-like growth factor (IGF) 1 and 2. Although studies have suggested a tumor-suppressive role for IGFBP-3, ample evidence in various cancers indicates that its high levels were associated with disease aggressiveness [29]. In a recent study on hepatocellular carcinoma, IGFBP3 was found highly expressed associated with disease poor prognosis [30]. By analyzing TCGA and GTEx data, we observed a significant IGFBP3 increase in HGG compared to normal brain tissues. However, IGFBP3 gene expression was also higher in LGG than in normal controls. It may be considered that IGFBP3 stimulates the tumorigenesis in both LGG and HGG probably via different pathways.

## Conclusions

As a conclusion, we identified robust differentially expressed genes associated with HGG and highlighted some important features of HGG tumors such as inflammation and stemness. These findings could enhance our knowledge about the molecular mechanisms underlying HGG tumors and provided reliable biomarkers for novel prognostic and therapeutic targets.

## Data Availability

The datasets used and/or analyzed during the current study are available from the corresponding author on reasonable request.

## Abbreviations

WHO: World health organization
LGG: Low-grade gliomas
HGG: High-grade gliomas
GBM: Glioblastoma multiform
IDH: Isocitrate dehydrogenase
GEO: Gene Expression Omnibus
TCGA: The Cancer Genome Atlas
DEGs: Differentially expressed genes
GO: Gene Ontology
KEGG: Kyoto Encyclopedia of Genes and Genomes
DAVID: Database for Annotation, Visualization, and Integrated Discovery
STRING: Search Tool for the Retrieval of Interacting Genes/Proteins
PPI: Protein–protein interaction
GEPIA: Gene Expression Profiling and Interactive Analyses
GTEx: Genotype-Tissue Expression
RT-qPCR: Reverse transcription‑quantitative polymerase chain reaction
EIF2B1: Eukaryotic translation initiation factor 2B subunit alpha
CTBP1: C-terminal binding protein 1
MRPL19: Mitochondrial ribosomal protein L9
TBP: TATA box-binding protein
PCA: Principal component analysis
MF: Molecular function
BP: Biological process
CC: Cellular component
ECM: Extracellular matrix
PTX3: Pentraxin 3
CHI3L1: Chitinase-3-like protein 1
IGFBP3: Insulin-like growth factor-binding protein 3
TIMP1: Tissue inhibitor of matrix metalloproteinases 1
LTF: Lactotransferrin
CEBPD: CCAAT / enhancer-binding protein delta
PDGFA: Platelet-derived growth factor subunit A
IGF: Insulin-like growth factor
TPM: Transcripts per million
